# Biological Activities of Secondary Metabolites from the Edible-Medicinal Macrofungi

**DOI:** 10.3390/jof10020144

**Published:** 2024-02-11

**Authors:** Xiaoqi Sun, Ying Shi, Dongxiao Shi, Yu Tu, Ling Liu

**Affiliations:** 1 State Key Laboratory of Mycology, Institute of Microbiology, Chinese Academy of Sciences, Beijing 100101, China; 2 University of Chinese Academy of Sciences, Beijing 100049, China

**Keywords:** macrofungi, mushrooms, secondary metabolites, biological activities

## Abstract

Macrofungi are well-known as edible-medicinal mushrooms, which belong mostly to Basidiomycota, with a few from Ascomycota. In recent years, macrofungi have been recognized as a rich resource of structurally unique secondary metabolites, demonstrating a wide range of bioactivities, including anti-tumor, antioxidant, anti-inflammatory, antimicrobial, antimalarial, neuro-protective, hypoglycemic, and hypolipidemic activities. This review highlights over 270 natural products produced by 17 families of macrofungi covering 2017 to 2023, including their structures, bioactivities, and related molecular mechanisms.

## 1. Introduction

Macrofungi, a kind of large and visible fungi, can form fleshy or gelatinous macroscopic fruiting bodies or sclerotia, which are usually known as mushrooms [1]. Most mushrooms belong to the Basidiomycete (such as *Lentinus edodes*), and a small portion belong to the Ascomycete (such as *morels* and *truffles*) [2]. These fruiting bodies contain a variety of nutrients, including protein, essential fatty acids, vitamins, and minerals [1]. Therefore, mushrooms are deeply loved by people due to their nutritional value. In order to protect the fruiting body from harmful organisms, including viruses, bacteria, and insects, mushrooms can secrete various secondary metabolites (such as terpenoids and phenolics) with antiviral, antioxidant, and other biological activities as chemical weapons. Mushrooms contain abundant active proteins, such as lectins with an antiviral effect [3], deoxyribonuclease with an anti-tumor function [4], and ribotoxin with an anti-proliferative effect [5]. Similarly, mushrooms are also one of the main sources of antioxidants ergothioneine and glutathione [6]. Modern pharmacology found that the secondary metabolites produced by mushrooms have certain medicinal value, including anti-Alzheimer [7], antidiabetic [8], and antitumor [9]. Lentinan is a type of glucan isolated from the fruiting body of shiitake mushrooms. It has detoxification activity and can significantly alleviate side effects such as leukocyte suppression, nausea, and vomiting caused by cancer chemotherapy. Currently, lentinan is used as injections for adjuvant treatment of cancer in clinical practices [10]. Additionally, lentinan can be used in combination with rabies vaccines. It not only resolves vaccine-induced side effects, but it also enhances therapeutic effects [11]. The mushroom resources that can be developed and utilized are very abundant. At the present time, approximately 2000 species of edible mushrooms and 650 species of medicinal mushrooms have been found in forests around the world [12], which have attracted the interest of food and pharmacy scholars due to their rich nutritional and medicinal values, as well as the abundant resources.

**Figure 1:**
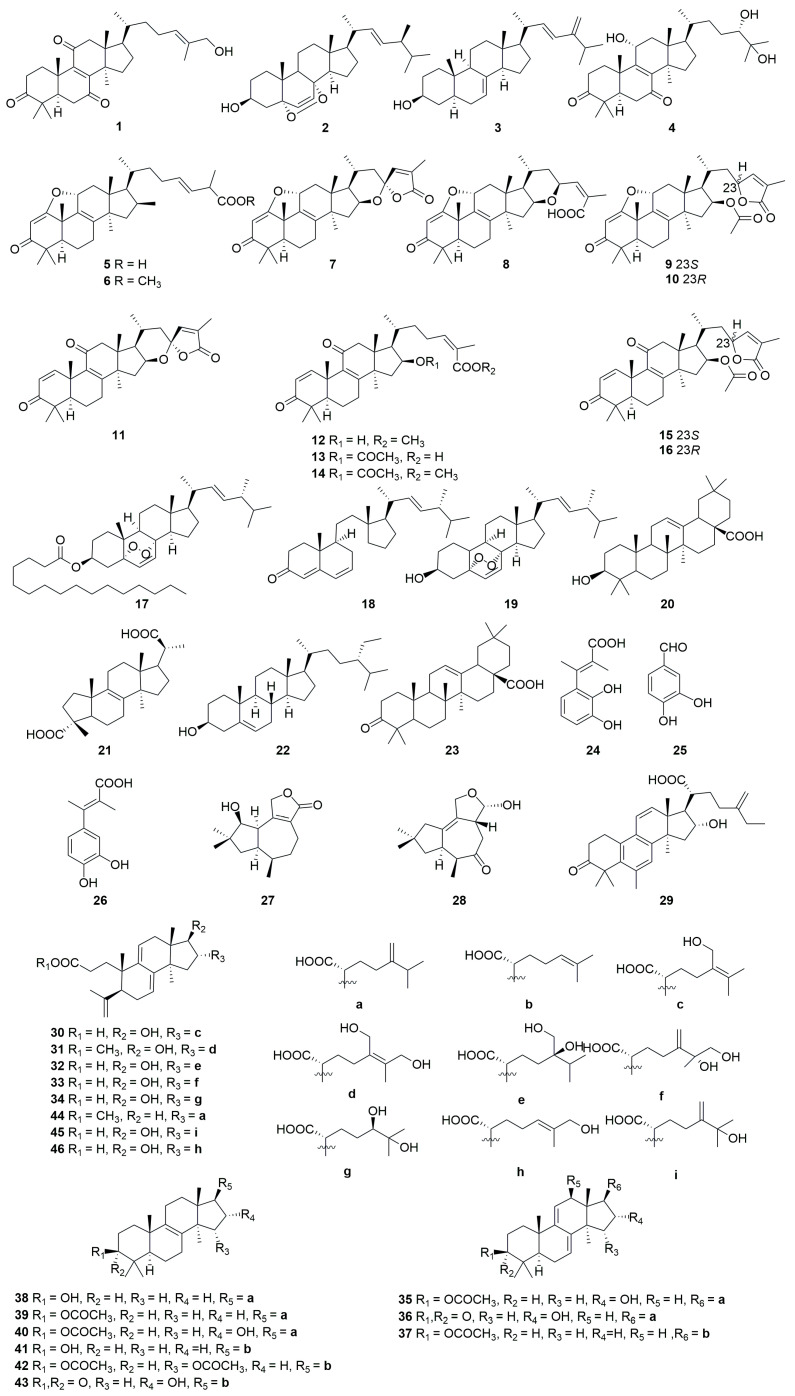
Structures of compounds **1**–**46**.

**Figure 2:**
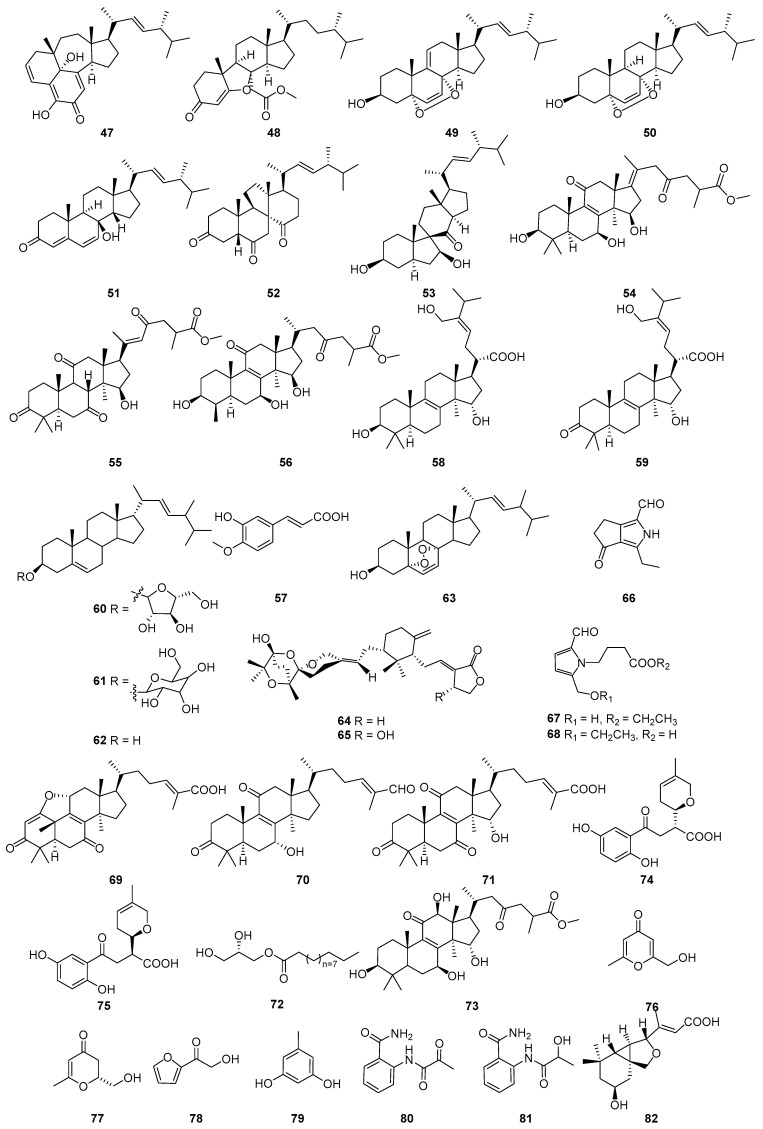
Structures of compounds **47**–**82**.

**Figure 3:**
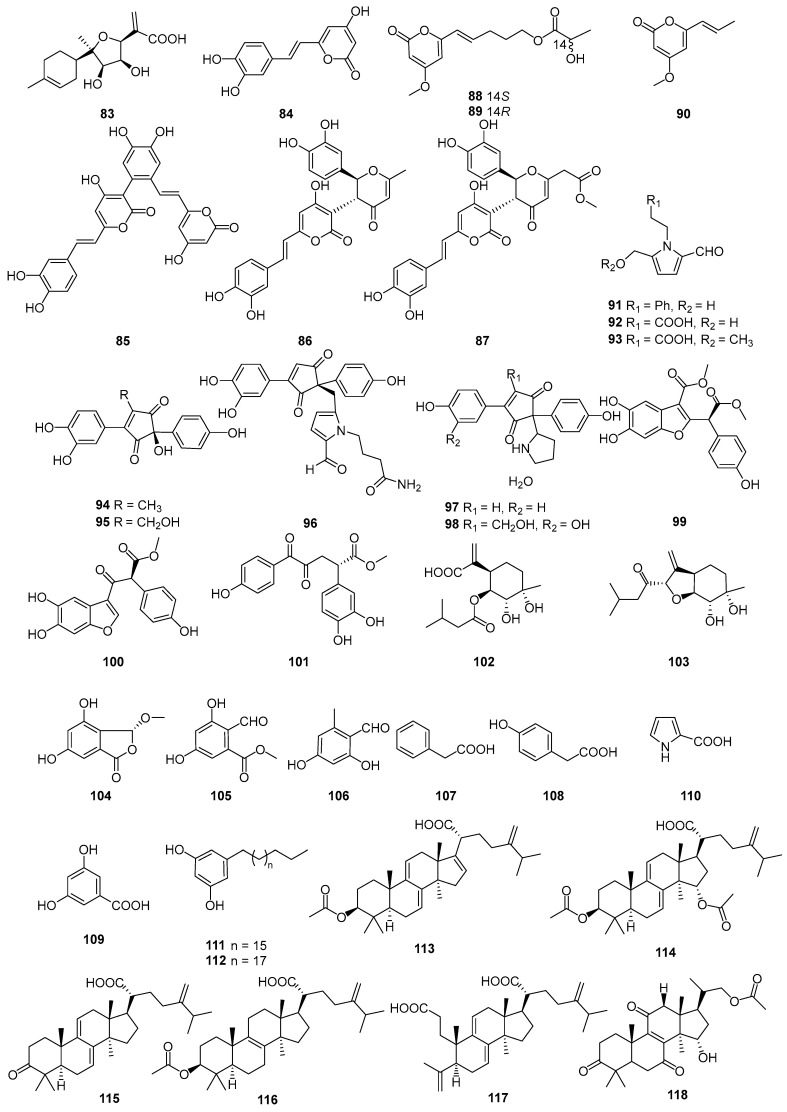
Structures of compounds **83**–**118**.

**Figure 4:**
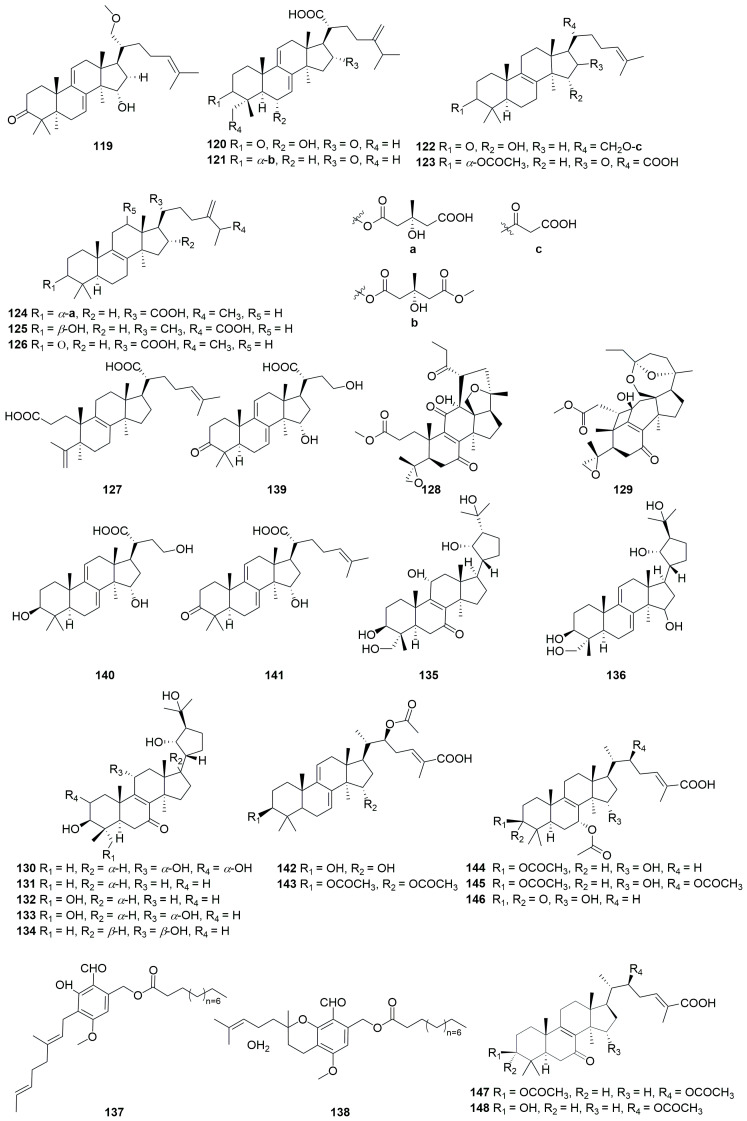
Structures of compounds **119**–**148**.

**Figure 5:**
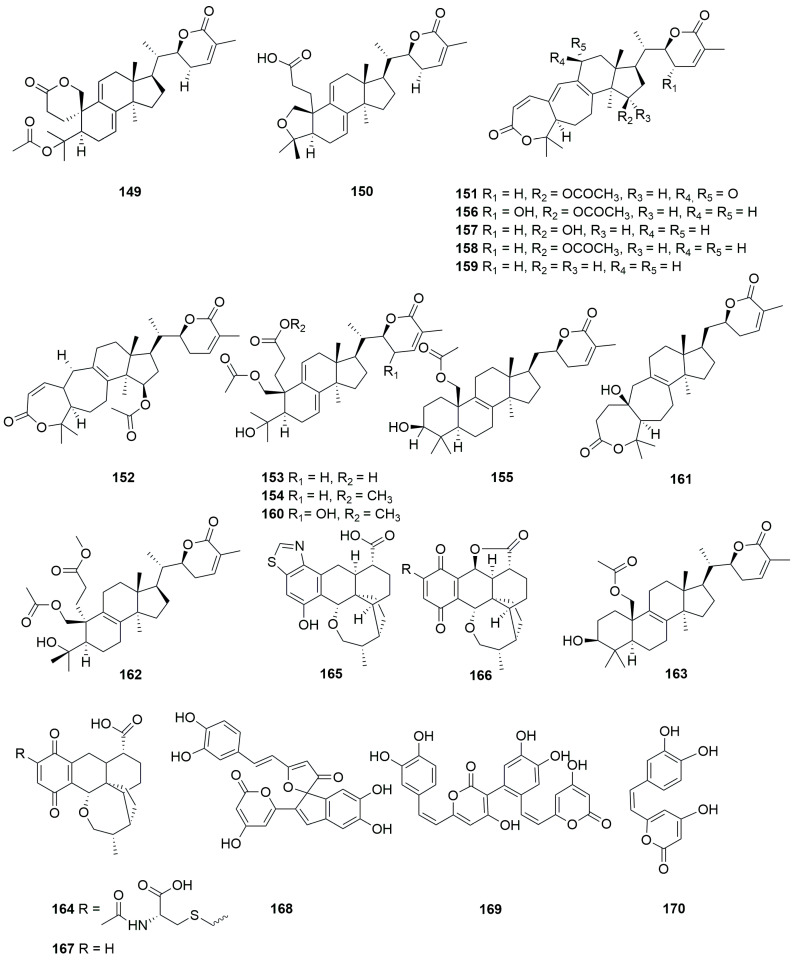
Structures of compounds **149**–**170**.

**Figure 6:**
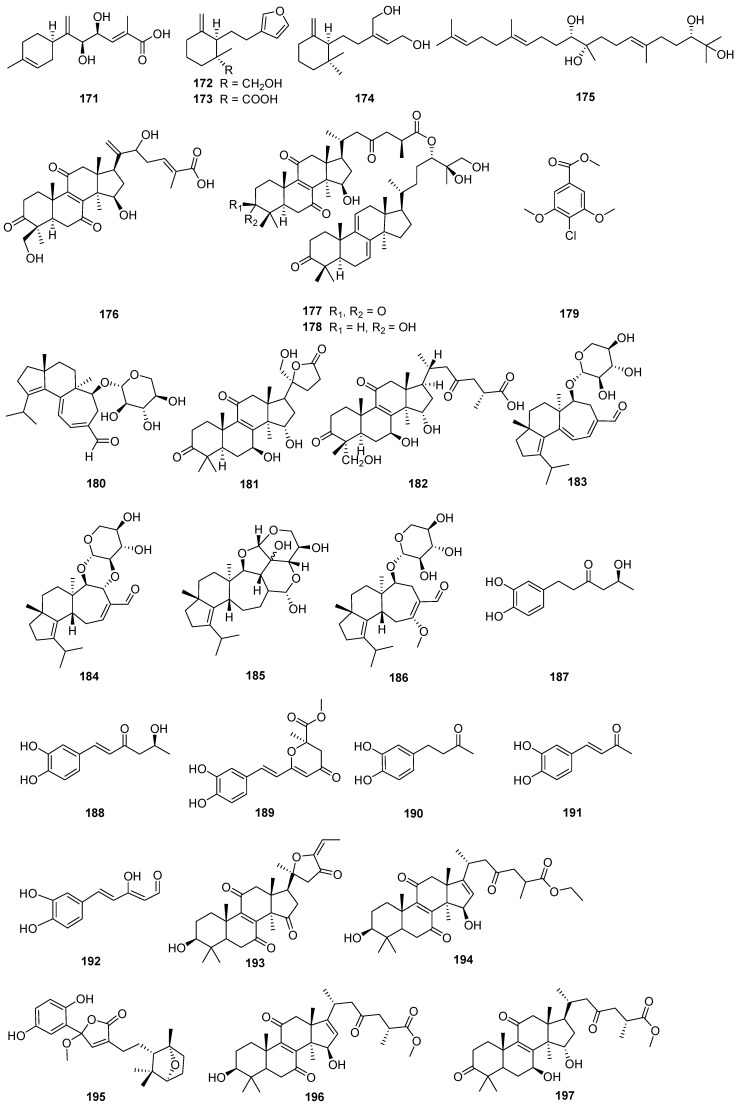
Structures of compounds **171**–**197**.

**Figure 7:**
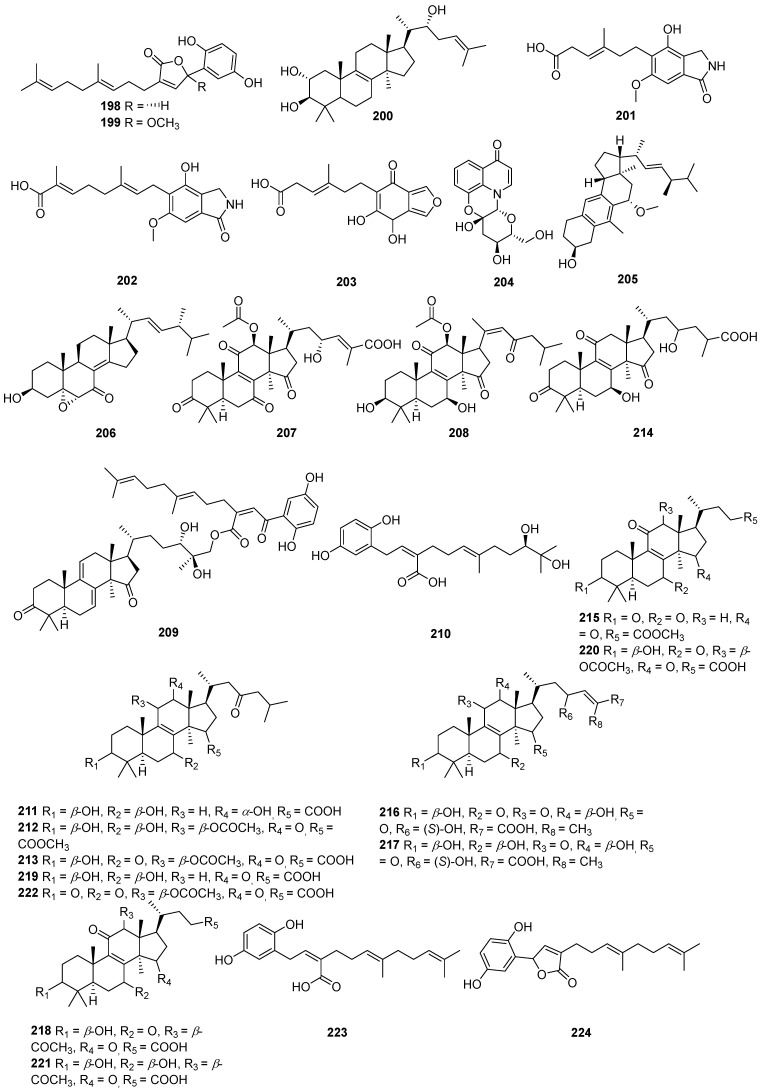
Structures of compounds **198**–**224**.

**Figure 8:**
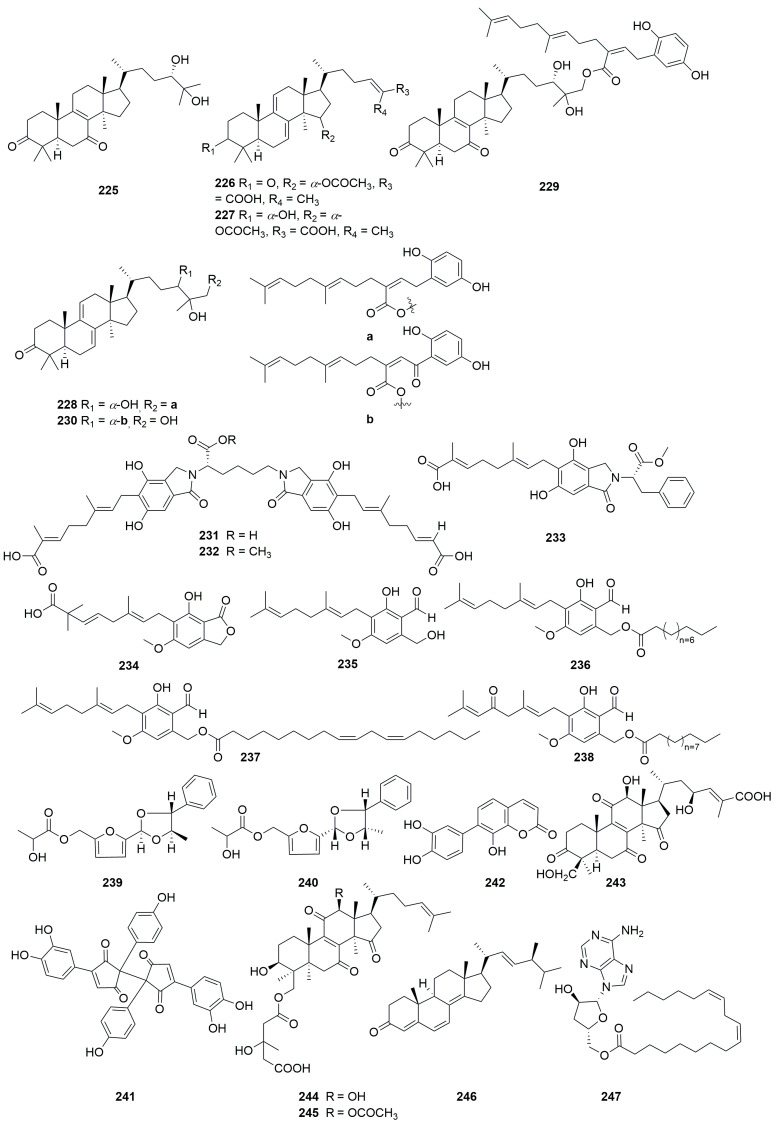
Structures of compounds **225**–**247**.

**Figure 9:**
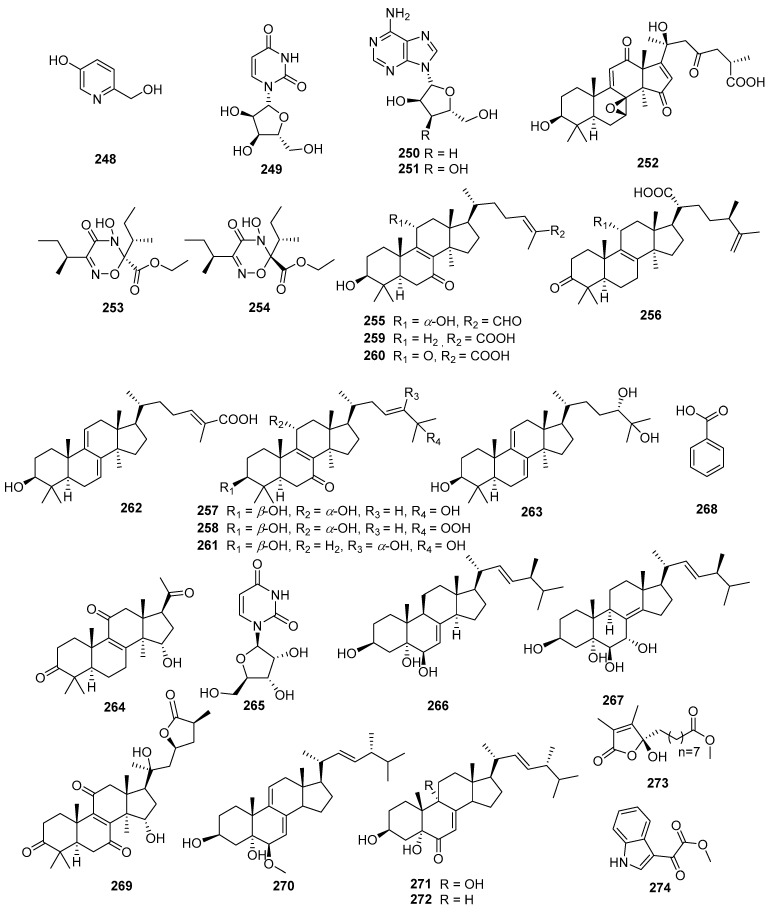
Structures of compounds **248**–**274**.

A comprehensive literature search of studies published from 2017 to 2023 was conducted by using the keywords “edible-medicinal mushroom”, “edible-medicinal macrofungi”, “edible mushroom”, “edible macrofungi”, “medicinal mushroom”, “medicinal macrofungi”, “mushroom”, “macrofungi” and “secondary metabolite”, and “secondary product” on PubMed, Web of Science, ACS, RSC, Reaxys, EMBASE, Springer Link, Elsevier Science Direct and Wiley. In this review, we focus on the latest progress of the biological activities of 274 secondary metabolites (Figure 1, Figure 2, Figure 3, Figure 4, Figure 5, Figure 6, Figure 7, Figure 8 and Figure 9) derived from macrofungi (Table 1). The aim of this review is to provide the theoretical basis for the research of secondary metabolites from large edible and medicinal fungi.

## 2. Biological Activities of Secondary Metabolites

### 2.1. Anti-Tumor Activity

The cancer incidence rate and cancer-related mortality are rising in the whole world. Based on cancer prevalence data, it is estimated that 28.4-million cancer cases will be diagnosed by 2040 [75]. At present, there are various methods of cancer treatment (such as chemotherapy, radiotherapy, immunotherapy, and others), among which chemotherapy is the most widely used [76]. Although chemotherapy drugs exhibit significant therapeutic effects, they have the disadvantages of low bioavailability, prominent side effects, and high susceptibility to drug resistance. In the last few years, it has been reported in the literature that natural active ingredients exhibit significant activity and few side effects [77], which have the potential as therapeutic drugs for inhibiting and treating cancer [13].

The following is a systematic review of the direct cytotoxic effect of secondary metabolites obtained from macrofungi fruiting bodies or mycelium against cancer cells. These studies mainly validated the inhibitory activity of secondary metabolites on malignant tumors such as lung cancer, liver cancer, and cervical cancer through 3-(4,5-dimethylthiazol-2-yl) -2,5-diphenyltetrazolium bromide (MTT) experiments.

The first example is a research evaluating the effect of secondary metabolites on human lung cancer cell lines using cell proliferation and cytotoxicity assays. The results demonstrated the suppressive activity of **2**, **3**, and **72** against A549, H1264, H1299, and Calu-6 cells. Among them, **3** exhibited the best suppressive activity on Calu-6 cells, with the half maximal inhibitory concentration (IC_50_) of 133.1 ± 4.6 μM [13]. Compound **4** (IC_50_ = 37.5–150 μM) displayed obvious antiproliferative and cytotoxic activity to MCF-7, HepG2, HeLa, HCT-116, and Caco-2 cells [14]. The results of MTT displayed that **11**–**16** had inhibitory activity against HL-60, A-549, SMMC-7721, MCF-7, and SW480 cell lines. Compounds **11** (IC_50_ = 3.52 ± 0.79−12.95 ± 0.73 μM), **12** (IC_50_ = 2.88 ± 0.08−8.27 ± 0.12 μM), and **16** (IC_50_ = 3.09 ± 0.03−12.48 ± 0.5 μM) exhibited more significant cytotoxicities, while **13** (IC_50_ = 10.77 ± 0.85−25.89 ± 0.87 μM), **14** (IC_50_ = 3.52 ± 0.79−12.95 ± 0.73 μM), and **15** (IC_50_ = 14.09 ± 0.86−23.23 ± 0.88 μM) exhibited moderate cytotoxicities (cisplatin as positive drug, IC_50_ = 2.95 ± 0.13−23.64 ± 1.6 μM) [15]. Compound **20** showed a cytotoxic effect against PC-3 with an IC_50_ value of 27.43 ± 0.86 µg/mL (doxorubicin as a positive drug, IC_50_ = 1.38 ± 0.16 μg/mL). Compounds **18**, **23,** and **24** were cytotoxic against MCF-7 with IC_50_ values of 28.74 ± 0.36, 8.90 ± 0.27, and 17.05 ± 0.54 µg/mL, respectively (cycloheximide as a positive drug, IC_50_ = 0.073 ± 0.12 μg/mL). Among them, **23** had the strongest antiproliferative activity against the MCF-7 cell line [16]. Compounds **27** and **28** showed obvious cytotoxicity against HL-60, SMMC-7721, and SW480 cell lines, especially on SW480 cells with IC_50_ values of 0.7 and 1.1 μM, respectively [17]. Compounds **36**, **37**, **39**, and **41** showed cytotoxicity against K562 cells, with IC_50_ values of 68.2 ± 2.2, 45.3 ± 1.2, 33.1 ± 0.5, and 25.7 ± 1.7 μM, respectively (cisplatin as a positive drug, IC_50_ = 3.8 ± 0.2 μM) [18]. Compound **47** showed obvious cytotoxic activity against HL-60, A-549, SMMC-7721, MCF-7, and SW480 cells, with the best suppressive activity on SW480 of the IC_50_ value of 18.1 μM [19]. Compounds **48**–**52** showed cytotoxicity against MCF-7 at a concentration of 100 μM [20]. Compound **53** exhibited a significant cytotoxic effect on HepG2 (IC_50_ = 9.1 μM) compared to the positive control sorafenib (IC_50_ = 5.5 μM). Moreover, using cisplatin with the IC_50_ value of 1.6 μM as the standard, **53** was evidently cytotoxic to the sorafenib-resistant Huh7 (IC_50_ = 6.2 μM) [21]. Compound **54** (IC_50_ = 6.82 ± 0.77 μM) had a cytotoxic effect on HGC-27 cells. Compounds **55** (IC_50_ = 13.67 ± 1.04 μM) and **56** (IC_50_ = 37.93 ± 3.22 μM) could inhibit the proliferation of A549 cells [22]. Compounds **60**–**63** had an inhibitory effect on HT29 and MCF7 cells, and **63** (IC_50_ = 38.8 ± 0.9 μg/mL) had a stronger effect on the HT29 cell line than the MCF7 cells [25]. The results of cell proliferation assay showed that **64** (IC_50_ = 1.5 ± 0.1−10.3 ± 0.25 μM) and **65** (IC_50_ = 0.3 ± 0.05−4.5 ± 0.15 μM) had cytotoxicity against HL-60, A-549, HepG2, Caki-1, and MCF-7 cells, which illustrated that **65** had stronger inhibitory activity (cisplatin as positive drug, IC_50_ = 1.2 ± 0.3−17.6 ± 0.4 μM) [26]. Compound **66** showed cytotoxicity against the SMMC-7721 cell line (IC_50_ = 15.8 μM) [27]. Compound **70** (IC_50_ = 17.1 μM) exhibited cytotoxicity against HL-60, while compound **1** had a moderate inhibitory effect on HL-60, SMMC-7721, A549, MCF-7, and SW-480 cells [28].

According to the above analysis, it is found that secondary metabolites derived from mushrooms have a significant anticancer effect, especially in inhibiting the growth of lung cancer cells (Calu-6), colorectal cancer cells (SW480), and hepatoma cells (SMMC-7721). Furthermore, it is worth mentioning that **27**, **28**, and **65** exhibit remarkable inhibitory activity against various cancer cells.

### 2.2. Antioxidant Activity

The pathogenesis of many human diseases (cancer, atherosclerosis, Alzheimer’s disease, and others) is related to oxidative stress. The occurrence of oxidative stress is caused by the imbalance between oxidation and antioxidation in the body [78]. Therefore, maintaining oxidative balance in the body helps to suppress the progression of diseases, and it is necessary to explore new drugs with antioxidant activity. The following is a general overview of studies on secondary metabolites with antioxidant activity of large edible and medicinal fungi in recent years. These studies mainly validated the antioxidant activity through 1,1-dipheny-1-2-picrylhydrazyl (DPPH) and 2,2-azinobis (3-ethylbenzothiazoline-6-sulphonic acid) (ABTS), the free-radical scavenging assay, the cupric-reducing antioxidant capacity (CUPRAC), and the oxygen radical-absorbance capacity [79].

The results of the ABTS assay showed that aromatic meroterpenoid compounds **74** (EC_50_ (concentration for 50% of maximal effect) = 0.59 ± 0.15 mM) and **75** (EC_50_ = 0.27 ± 0.05 mM) had stronger radical scavenging effects than the positive drug (trolox, EC_50_ = 0.42 ± 0.03 mM). Moreover, ORAC experimental results indicated that **74** (5.42 ± 0.2 µmol TE/µmol) and **75** (7.24 ± 0.15 µmol TE/µmol) had strong antioxidant activity. Among them, the antioxidant effect of **75** was similar to the positive control (quercetin 7.78 ± 0.27 µmol TE/µmol) [29]. ABTS, DPPH, and *β*-carotene linoleic acid experiments results showed that phenolic compounds **25** (IC_50_ = 1.06 ± 0.1−3.14 ± 0.11 μg/mL) and **26** (IC_50_ = 1.06 ± 0.46−10.26 ± 0.56 μg/mL) exhibited antioxidant activity [16]. In the DPPH radical-scavenging experiment, Vitamin C was used as the positive control. Compound **78** had strong scavenging ability at the concentration of 0.25 mg/mL, while **76** and **77** had moderate scavenging ability against DPPH radical [30]. Compound **79** had a scavenging ability against DPPH radicals, while **80** and **81** had less scavenging ability against DPPH radicals [31]. The DPPH radical-scavenging activity of **57** (IC_50_ = 21.7 μg/mL) was better than that of Vitamin C (IC_50_ = 50.0 μg/mL) [23]. Compound **68** showed antioxidant activity at the concentration value of 50 μM against the DPPH radical [27]. Moreover, it was found that phenolic compounds (**84**–**87**) had good antioxidant activity in a dose-dependent manner. And the antioxidant activity of **86** was the best, with the cellular antioxidant activity value of 5.31 μM [32]. Compounds **88** (IC_50_ = 10.39 ± 2.26−18.58 ± 2.33 μg/mL) and **89** (IC_50_ = 16.57 ± 2.48−20.43 ± 3.74 μg/mL) showed significant radical-scavenging activities [33]. An analysis of the results of antioxidant experiments revealed that **96** (IC_50_ = 11.5 ± 0.51 μM) and **98**–**101** (IC_50_ = 6.7 ± 0.05−15.5 ± 0.50 μM) had stronger activity against the DPPH radical than the positive drug (tert-butylhydroquinone, IC_50_ = 17.3 ± 1.32 μM). Moreover, Compounds **94**–**101** (IC_50_ = 44.6 ± 5.12−186.7 ± 8.14 μM) had the ability to scavenge superoxide anion radicals, and compounds **96**–**101** (IC_50_ = 99.5 ± 11.27−292.7 ± 12.5 μM) had ability to scavenge hydroxyl radicals than the positive drug (tert-butylhydroquinone, IC_50_ = 186.7 ± 8.14 μM). In the meanwhile, compounds **94** (IC_50_ = 783.4 ± 20.35 μM), **95** (IC_50_ = 897.9 ± 26.39 μM), **97** (IC_50_ = 667.2 ± 12.17 μM), and **101** (IC_50_ = 292.7 ± 12.5 μM) showed a weak ability to scavenge hydroxyl radicals (tert-butylhydroquinone as positive drug, IC_50_ = 271.5 ± 19.22 μM) [34]. Compound **103** (EC_50_ = 573 μM) showed moderate activity against the DPPH radical [35]. Phenolic compounds (**104**–**110**) showed a DPPH radical scavenging ability, and the IC_50_ value of these compounds were 1.79, 4.10, 4.28, 2.45, 4.40, 1.73, and 6.00 mM, respectively [36]. In the ABTS radical-scavenging activity test, **111** (EC_50_ = 0.001 mg/mL) and **112** (EC_50_ = 0.145 mg/mL) showed strong antioxidant activity compared with Vitamin C (EC_50_ < 0.025 mg/mL) [37].

Based on the above analysis, it is found that aromatic meroterpenoid and phenolic compounds derived from mushrooms have obvious antioxidant activities, especially phenolic compounds. To sum up, **25**, **26**, **57**, **78**, **86**, **96**, **98**–**101**, **109**, **111,** and **112** exhibit outstanding a radical scavenging activity.

### 2.3. Anti-Inflammatory Activity

Inflammation is a protective response against injury and infection by secreting nitric oxide (NO) and pro-inflammatory cytokines [80]. A moderate inflammatory response helps to resist external harmful stimuli. However, excessive inflammation can easily lead to acute diseases such as enteritis and arthritis. And prolonged inflammation can easily cause chronic diseases [81]. Drugs including steroids and nonsteroidals are commonly used in clinical to alleviate inflammation even though their side effects (hypertension, hepatotoxicity, and others) are non-negligible [82]. Therefore, it is imperative to search for potential low-toxic and effective anti-inflammatory drugs. The following is a summary of studies on secondary metabolites with the anti-inflammatory activity of large edible and medicinal fungi in recent years. These studies mainly validated the anti-inflammatory activity through constructing inflammation models using lipopolysaccharide (LPS).

Compounds **5**–**10** (IC_50_ = 17.9–34.9 μM) could prominently inhibit the production of NO by LPS-induced RAW264.7 macrophages in mice and were superior to L-N^G^ monomethylarginine (IC_50_ = 47.1 μM) [15]. Evaluated by the same experiment, Compounds **69**–**72** had anti-inflammatory properties at lower concentrations (6.25–25 μM) [28]. Compounds **113**–**117** (IC_50_ = 49.43–82.32 μM) exhibited potential anti-inflammatory activity and could strongly inhibit the production of NO in RAW264.7 macrophages; compound **117** (IC_50_ = 49.43 μM) had the strongest activity. Further study on the mechanism showed that **117** inhibited the expression of nuclear factor-kappa B (NF-*κ*B), nitric oxide synthase (iNOS), and cyclooxygenase-2 (COX-2) to exert anti-inflammatory effect [38]. In the LPS-induced inflammatory response of RAW264.7 macrophages, **118** (IC_50_ = 38.6 ± 1.0 μM) considerably inhibited the release of NO, as well as pro-inflammatory cytokines (interleukin-1*β* (IL-1*β*) and interleukin-6 (IL-6)) together with the phosphorylation of inhibitor kappa B kinase *β* (IKK*β*) and inhibitor kappa B alpha (IκBα). These results indicated that **118** inhibited the NF-*κ*B-signaling pathway to exert anti-inflammatory activity [39]. Compounds **119**–**126** could inhibit the NO released from the LPS-induced RAW264.7 macrophages, with IC_50_ values in the range of 21.4–27.2 μM. Among them, **126** (IC_50_ = 21.4 ± 0.04 μM) exhibited better anti-inflammatory activity (hydrocortisone as a positive drug, IC_50_ = 22.4 ± 1.56 μM) [40]. Compounds **128** (IC_50_ = 20.77 μM) and **129** (IC_50_ = 18.52 μM) had strong anti-inflammatory activity and could suppress the NO released from the LPS-induced BV-2 microglial cells [41]. Compounds **130**, **131**, **134**, and **136** could notably inhibit the NO released from the LPS-induced BV-2 microglial cells with IC_50_ values ranging from 2.32 to 23.83 μM. Among them, **130** (IC_50_ = 3.985 ± 0.01 μM), **131** (IC_50_ = 4.074 ± 0.03 μM)**,** and **134** (IC_50_ = 2.32 ± 0.02 μM) had better anti-inflammatory activity and were superior to positive drugs (quercetin, IC_50_ = 4.01 ± 0.7 μM) [42]. Compounds **137** (IC_50_ = 56.33 ± 6.81–87.31 ± 8.77 μM) and **138** (IC_50_ = 48.50 ± 6.54−76.16 ± 9.11 μM) inhibited the release of pro-inflammatory mediators (tumor necrosis factor-*α* [TNF-*α*], IL-6, and NO) in LPS-induced RAW 264.7 macrophage cells in a dose-dependent manner (aspirin as positive drug, IC_50_ = 27.08 ± 1.86−51.82 ± 8.62 μM) [43]. Compounds **139**–**141** (IC_50_ = 14.3–42.3 μM) could significantly inhibit the production of NO by the LPS-induced RAW264.7 cell line and were superior to minocycline (IC_50_ = 73.0 μM) [44]. The anti-inflammatory experimental results showed that **142** (IC_50_ = 0.9 ± 0.1 μM) and **143** (IC_50_ = 0.6 ± 0.1 μM) prominently reduced the secretion of NO at extremely low doses and completely suppressed the release of TNF-*α* and IL-6 at 10 μM (silymarin as a positive drug, IC_50_ = 1.8 ± 0.4−86.5 ± 2.5 μM) [45].

As to the analysis above, it is found that these compounds exert an anti-inflammatory effect by inhibiting the secretion of NO and the expression of IL-6 and TNF-*α* and the NF-*κ*B-signaling pathway. Meanwhile, Compounds **69**–**72**, **126**, **129**–**131**, **134**, and **143** have remarkable anti-inflammatory effects. Therefore, it is speculated that these secondary metabolites have the potential to become highly effective anti-inflammatory agents.

### 2.4. Antimicrobial Activity

Invasive fungal and bacterial infections are key causes of the incidence rate and mortality in immune-compromised populations [83,84]. Antibiotics are often used for the treatment of microbe infections. However, the emergence of antibiotic resistance has greatly reduced the effectiveness of antimicrobial drugs and will increase the severity of infection, morbidity, and treatment costs [85]. Therefore, newer and more effective antibiotics are urgently needed. Similarly, the treatment cycle for fungal infection is long, which means there is an urgent need to explore more effective antifungal drugs. The following is a systematic review of studies on secondary metabolites with the antimicrobial activity of mushrooms in recent years. These studies mostly evaluated the inhibitory activity of secondary metabolites on various fungi (*Candida albicans* and *Cryptococcus neoformans*) and bacteria (*Mycobacterium tuberculosis* and *Staphylococcus aureus*) through the minimum inhibitory concentration (MIC) index. Moreover, in order to facilitate the comparison of the strength of activity of the compounds, the MIC values were categorized into three classes according to Kuete’s work [86]: significant (MIC < 10 μg/mL), moderate (10 < MIC < 100 μg/mL), and low or negligible (MIC > 100 μg/mL).

Compared with the positive drug (isoniazid, MIC = 0.047 μg/mL), **13** (MIC = 50 μg/mL) was moderately active in inhibiting the growth of *M. tuberculosis* H37Ra [15]. The results of the antimicrobial activity experiment showed that **58** (MIC = 66 μg/mL) had moderate activity against the sensitive non-pathogenic zygomycete *Mucor hiemalis* [24]. Using ciprofloxacin (MIC = 0.9 μM) as the positive drug, **102** (MIC = 90.3 μM) exhibited moderate inhibitory activity against *S. aureus* [35]. Among the secondary metabolites obtained from *Ganoderma* species, 7*α*-acetoxy derivatives **144**–**146** (MIC = 12.5−25 μg/mL) exhibited moderate anti-tuberculosis activity, while **147** (MIC = 6.25 μg/mL) and **148** (MIC = 1.56 μg/mL) could significantly inhibit *M. tuberculosis* H37Ra growth (isoniazid as positive drug, MIC = 0.094 μg/mL) [46]. The results of the anti-tuberculosis activity experiment showed that compound **163** (MIC = 12.5 μg/mL) had moderate activity [47]. Furthermore, both **166** (MIC = 16.7–100 μg/mL) and **167** (MIC = 8.3–66.7 μg/mL) had shown a moderate inhibitory effect on fungi (*M. hiemalis*, *C. tenuis*) and Gram-positive bacteria (*Micrococcus luteus, S. aureus*, and *Bacillus subtilis*). Compounds **164** (MIC = 33.3–66.7 μg/mL) and **165** (MIC = 33.3–66.7 μg/mL) had a moderate inhibitory effect on some yeasts (*Pichia anomala* and *Rhodotorula glutinis*) [48]. Compounds **168** (MIC = 25 μg/mL) and **170** (MIC = 100 μg/mL) could inhibit the proliferation of *M. luteus* [49]. Compounds **171**–**174** (MIC = 75–100 μg/mL) exhibited moderate antibacterial activity (*S. aureus*), while **174** exhibited an inhibitory effect on both fungi (*M. hiemalis*) and bacteria (*S. aureus* and *B. subtilis*) [50]. Compound **175** (MIC = 0.9–3.1 μg/mL) could significantly inhibit the reproduction of human pathogenic fungi (*C. albicans* and *C. neoformans*), and its activity was equivalent to the amphotericin B (MIC = 0.4–0.8 μg/mL) [51].

Based on the above analysis, compounds **147** and **148** are the potential drugs for the treatment of tuberculosis.

### 2.5. Antimalarial Activity

Malaria is a disease caused by the infection of protozoan parasites in the genus *Plasmodium*, which is prevalent in tropical regions (especially in Africa, Southeast Asia, and South America) and has a significant mortality rate [87]. It is necessary to explore effective antimalarial drugs. The following is a brief review of studies on secondary metabolites with antimalarial activity of mushrooms in recent years. Compounds **149**–**162** (IC_50_ = 5.1−19 μM) from the fruiting body of the wood-rot *Tomophagus* sp. showed antimalarial activity, of which **150** (IC_50_ = 5.1 μM) had the strongest antimalarial activity (dihydroartemisinin as positive drug, IC_50_ = 0.0028 μM) [47]. Compound **176** (IC_50_ = 257.8 nM) had an inhibitory effect on chloroquine-sensitive strain *P. falciparum* (chloroquine as positive drug, IC_50_ = 22.66 nM) [52]. The antimalaria test results showed that **177** (IC_50_ = 0.05 μM) and **178** (IC_50_ = 0.45 μM) had significant inhibitory effects on *P. falciparum*, while chloroquine (IC_50_ = 0.50 μM) was used as positive control [53]. Overall, compounds **150**, **177**, and **178** exhibit potential antimalarial activity effects. It is speculated that these compounds have the potential to become more effective antimalarial drugs.

### 2.6. Neuro-Protective Activity

In recent years, the incidence rate of nervous system diseases (Alzheimer’s disease, stroke, and others) has increased continuously and has gradually become the main cause of global disability [88]. Neuroglial cells are the most important cells in the central nervous system, which maintain homeostasis and the operation of the central nervous system through interactions with neurons, immune cells, and other factors [89]. The following is a systematic review of studies on secondary metabolites with the neuroprotective effect of large edible and medicinal fungi in recent years. These studies mainly validated the neuroprotective effect by constructing neural system injury models using the PC12 cell line and BV-2 microglia.

Compounds **73**–**75** could alleviate H_2_O_2_, and amyloid *β*-protein-induced SH-SY5Y cells damaged through the reduction of the production of reactive oxygen species (ROS) or free radicals [29]. Compounds **128** and **129** could exert neuroprotective effects by reducing the production of NO in BV-2 microglia [41]. Compounds **130**–**136** exerted neuroprotective effects by reducing the production of NO in BV-2 microglia, with **130**, **131**, and **134** exhibiting superior neuroprotective activity compared to positive drugs (quercetin) [42]. Compounds **179** and **180** could enhance nerve growth factor-induced neurite outgrowth in PC12 cells through the tyrosine kinase A (TrkA) and kinase1/2 (ERK1/2) pathway to exert neuroprotective effect [54]. Compounds **181** and **182** could alleviate hydrogenperoxide (H_2_O_2_)-induced PC12 cell damage by stimulating neurite activity. Through electron microscopy observation, it was found that **181** and **182** showed neurotrophic effects on undifferentiated PC12 cells [55]. Compounds **183**–**186** exerted neuroprotective effects by promoting the release of neurotrophic factors in astrocytic cells [56]. Compounds **187**–**192** exhibited neurotrophic activity and could promote axonal growth [57]. Compounds **193**–**199** could exert neuroprotective effects by reducing the production of NO in BV-2 microglia, and **193** had the most significant effect. Further research found that the neuroprotective effect of **193** could be related to the reduction of the levels of IL-1*β*, IL-6, and TNF-*α* and the mitigation of abnormal changes in mitochondrial membrane potential and reduction in ROS generation. Mechanism studies showed that the neuroprotective effect of **193** was related to its inhibition of TLR-4/NF-*κ*B and MAPK-signaling pathways and activation of the Akt/GSK-3*β*/Nrf2-signaling pathway [58]. Compound **200** reduced the production of ROS and alleviated mitochondrial damage to protect SH-SY5Y cells from H_2_O_2_-induced damage, which could be related to the Nrf2- and BDNF/TrkB/ERK/CREB-signaling pathways [59]. Compounds **201**–**203** could considerably enhance neurite outgrowth in PC-12 cells [60]. Compounds **204**–**206** exerted a neuroprotective effect by reducing the levels of inflammatory cytokines. Compound **201** could markedly reduce the level of TNF-*α* in BV-2 microglia, and **205** significantly reduced the level of IL-6 in BV-2 microglia. Compound **206** noticeably reduced the levels of NO and IL-1*β* in BV-2 microglia, as well as the expression of phosphorylated nuclear factor-kappa B inhibitor-*α* and the activity of iNOS [61].

According to the above analysis, it is found that these compounds exert neuroprotective effects by mitigating oxidative stress, expressing pro-inflammatory factors, activating TrkA/ERK1/2-, Nrf2-, or BDNF/TrkB/ERK/CREB-signaling pathways, or inhibiting TLR-4/NF-*κ*B- or MAPK-signaling pathways.

### 2.7. Hypoglycemic Activity

The elevation of blood glucose levels mainly causes diabetes, which also causes metabolic disorders in the organism. Moreover, high blood glucose can easily induce cardiovascular diseases, obesity, kidney diseases, and other diseases [90]. Therefore, maintaining blood glucose balance in the body is of great importance. The *α*-glucosidase, aldose reductase (AR), maltase, sucrase, and protein tyrosine phosphatase 1b (PTP1B) play important roles in lowering blood glucose levels [91,92,93,94]. The *α*-glucosidase and AR can mainly act on hyperglycemia to lower blood glucose levels [95,96]. PTP1B can reduce blood glucose levels by negatively regulating the insulin metabolism pathway [97]. This section reviewed promising drugs derived from mushrooms that can suppress *α*-glucosidase, AR, maltase, sucrase, and PTP1B enzyme activities.

Compounds **29**–**36** and **38**–**45** exhibited insulin-sensitization effects, and compound **46** significantly increased glucose uptake in 3T3-L1 adipocytes [18]. Compounds **82**–**87** could effectively alleviate insulin-induced decreases in glucose uptake in HepG2 cells and **82** had the minimum glucose uptake rate of 36.6% [32]. Compounds **121**–**125** and **127** had significant inhibitory effects on PTP1B, with IC_50_ values in the range from 20.5 to 56.4 μM. Compounds **123**, **124**, and **127** could effectively improve the decrease of glucose uptake in HepG2 cells induced by insulin [40]. Compounds **207**, **208**, **210**–**212**, **215**, **216**, and **222**–**229** had prominent suppressed effects against AR. Compound **224** (IC_50_ = 19.1 μM) had similar suppressed activity on AR as positive control epalrestat (IC_50_ = 17.5 μM). Compounds **207** (IC_50_ = 60.1 ± 10.1 μM), **209** (IC_50_ = 91.2 ± 13.6 μM), **210** (IC_50_ = 18.1 ± 2.3 μM), **218** (IC_50_ = 46.5 ± 5.1 μM), **220** (IC_50_ = 32.5 ± 3.1 μM), **223** (IC_50_ = 5.4 ± 0.4 μM), **224** (IC_50_ = 0.6 ± 0.12 μM), **228** (IC_50_ = 21.5 ± 2.2 μM), and **230** (IC_50_ = 8.1 ± 2.9 μM) had a suppressed effect against *α*-glucosidase, while acarbose (IC_50_ = 38.1 ± 6.0 μM) was used as positive control. Moreover, Compounds **207** (IC_50_ = 51 ± 3.5 μM), **210** (IC_50_ = 11.1 ± 0.3 μM), **218** (IC_50_ = 52.1 ± 9.1 μM), **220** (IC_50_ = 16.9 ± 1.2 μM), **223** (IC_50_ = 17.1 ± 4.2 μM), **224** (IC_50_ = 3.9 ± 0.7 μM), and **230** (IC_50_ = 10.1 ± 1.9 μM) had inhibitory effects against maltase, while acarbose (IC_50_ = 16.1 ± 4.1 μM) was used as a positive control. Compounds **211**−**215** (IC_50_ = 18.1 ± 2.5−41.9 ± 4.9 μM), **219** (IC_50_ = 7.6 ± 2.8 μM), and **220** (IC_50_ = 25.8 ± 3.1 μM) showed suppressed activity against PTP1B, while sodium vanadate (IC_50_ = 1.3 ± 0.2 μM) was used as a positive control [62]. Compounds **231**–**233** (IC_50_ = 36.2−40.8 μM) displayed good *α*-glucosidase inhibitory activity [63]. Compounds **234**–**238** (IC_50_ = 3.9−19.6 μM) exhibited significant inhibition of *α*-glucosidase, and **237** (IC_50_ = 3.9 μM) had better inhibitory activity, while acarbose (IC_50_ = 71.2 μM) was used as a positive control [64]. Similarly, the acarbose (IC_50_ = 59.48 ± 2.13 μM) was used as the positive drug, **239**–**242** exhibited significant inhibitory against *α*-glucosidase, and **241** had the best inhibitory activity (IC_50_ = 14.65 ± 1.68 μM) [65].

Based on the above analysis, it is found that **210**, **223**, and **224** have a significant suppression of *α*-glucosidase, AR, maltase, and sucrase. Additionally, compounds **35**, **38**, **40**, **82**, **123**, **124**, and **127** effectively inhibit cell absorption of glucose. Therefore, they have great potential in the treatment of diabetes.

### 2.8. Hypolipidemic Activity

The global prevalence of obesity is on the rise, and obesity is prone to induce a variety of diseases (diabetes, cardiovascular disease, and others) [98]. Lipases, especially pancreatic lipase, are essential enzymes for lipid absorption, and 3-hydroxy-3-methyl glutaryl Coenzyme A reductase (HMG-CoA) is a rate-limiting enzyme in cholesterol biosynthesis [99,100]. Therefore, HMG-CoA and lipase are important targets for treating obesity symptoms. This section reviewed secondary metabolites derived from mushrooms with inhibitory effects against HMG-CoA reductase and lipase enzyme. Using atorvastatin (IC_50_ = 32.1 μM) as the standard, **217** (IC_50_ = 29.8 μM), **221** (IC_50_ = 16.5 μM), **223** (IC_50_ = 30.3 μM), and **224** (IC_50_ = 14.3 μM) dramatically inhibited HMG-CoA [62]. Analyzing the experimental results of HMG-CoA activity, it was speculated that **243**–**245** were HMG-CoA reductase inhibitors and **245** (IC_50_ = 8.68 μM) had the best inhibitory activity against HMG-CoA, while atorvastatin (IC_50_ = 32.1 ± 7.7 μM) was used as a positive control [66]. Compared with the positive drug (orlistat), **246** exhibited inhibitory activity against pancreatic lipase [67]. Compounds **247**–**251** had prominent inhibitory activity on lipase [68]. In general, **224**, **246,** and **251** have great potential in the treatment of obesity.

### 2.9. Other Bioactivities

Edible and medicinal mushrooms can effectively produce useful bioactive metabolites. Due to their anti-tumor, anti-inflammatory, and antioxidant activities, these secondary metabolites have protective effects on organs such as the liver and kidneys. In addition, these secondary metabolites also have immunomodulatory activity and inhibitory effects on some cholinesterase and tyrosinase.

Compounds **17**, **18**, **20**, and **22**–**24** exhibited inhibitory effects on acetylcholinesterase (AChE) at the concentration of 100 μg/mL. Moreover, Compounds **20** and **23** exhibited inhibitory effects on butrylcholinesterase (BchE) at 100 μg/mL. Compounds **18**–**20**, **21**, **23**, **25**, and **26** exhibited a suppressed effect on tyrosinase at 100 μg/mL [16]. Compounds **66** and **67** exerted immunosuppressive activity by inhibiting an LPS-induced proliferation of T cells at 20 μM [27]. Compounds **88**–**93** (MIC = 25.66 ± 2.84−55.28 ± 3.34 μg/mL) exhibited a moderate inhibitory effect on tyrosinase [33]. The results of lymphocyte proliferation test and the Concanavalin A-induced T lymphocyte mitogenic activity test showed that **252** had immune activity at 0.1 μM [69]. Compounds **253** (IC_50_ = 6.61 μM) and **254** (IC_50_ = 10.67 μM) significantly suppressed B lymphocyte cell proliferation induced by LPS [70]. Compounds **255**–**264** could reduce the activities of alanine aminotransferase and aspartate aminotransferase at 5−20 μM in HepG2 cells to exert liver-protective activity [71]. Compounds **265**–**268** (10 μM) alleviated cisplatin-induced nephrotoxicity by inhibiting the JNK-caspase-3 pathway [72]. Compound **269** exhibited inhibitory effect against AchE at 50 μM [73]. Compounds **270**–**274** (IC_50_ = 60.47 ± 2.63−148.38 ± 23.67 μM) could inhibit tyrosinase activity, and **274** (IC_50_ = 60.47 ± 2.63 μM) had the strongest activity (arbutin, MIC = 58.17 ± 6.09 μM, was used as the standard) [74]. Therefore, **66** and **253** show the best immunosuppressive effect. Compounds **255**–**268** have a protective effect on the liver or kidney, while Compounds **20**, **269**, and **223** exhibit potential activities to be used as AchE or BchE inhibitors. Similarly, it is speculated that **26** and **274** can serve as tyrosinase inhibitory agents.

## 3. Discussion

As early as prehistoric times, there were records of mushrooms as medicines [101]. Due to the rich nutritional content of mushrooms, they have always attracted scholars to deeply research the mechanisms behind their medicinal properties [102]. At present, there is considerable research on mushroom polysaccharides, and it has been reported that the immune regulation and anti-tumor effect of mushrooms mainly stem from mushroom polysaccharides [103]. With the continuous deepening of research on the composition of mushrooms, the secondary metabolites produced from mushrooms have also attracted scholars’ attention.

According to further data analysis, 61 secondary metabolites show hypoglycemic activity, 42 metabolites exhibit anti-tumor function, 40 metabolites have neuroprotective effects, 19 metabolites are able to inhibit microorganisms, 13 metabolites have hypolipidemic activity, 18 metabolites display inhibitory tyrosinase activity, 11 metabolites exhibit enzyme-inhibitory activity, 3 metabolites possess insecticidal activity, and 6 metabolites exhibit organ-protective functions. Furthermore, it is worth mentioning that oxidative stress and inflammatory responses are often key pathological processes in some acute and chronic diseases (Alzheimer’s disease, heart failure, and others) [104,105,106]. There are 35 and 37 secondary metabolites with antioxidant and anti-inflammatory effects, respectively. And their mechanisms may relate to TrkA/ERK1/2, Nrf2, BDNF/TrkB/ERK/CREB, TLR-4/NF-*κ*B, and MAPK pathways. Summarizing the above, it can be hypothesized that mushrooms are important drug resources used to treat diabetes, cancer, neurological diseases, fungal and bacterial infectious diseases, and hyperlipidemia. However, it is undeniable that although these secondary metabolites reviewed in this paper have certain medicinal potential, further exploration is still needed to determine whether they produce side effects and have sufficient yield.

In addition, most of the edible and medicinal mushrooms included in this article come from *Ganodermataceae*, *Hymenochaetaceae*, *Polyporaceae*, and *Hericiaceae*, indicating that mushrooms from these families produce a wide range of pharmacologically active secondary metabolites. Overall, macrofungi is a rich source of bioactive secondary metabolites with a high potential for exploitation.

## 4. Conclusions

In recent years, relevant scholars have elaborated on the types of medicinal and edible mushrooms, the extraction techniques of bioactive components from mushrooms, and the pharmacological effects of active metabolites. The research on active ingredients focuses on primary metabolites (polysaccharides, proteins, polyunsaturated fatty acids) and nutritional components of mushrooms themselves, along with their development in food, medicine, and other fields [107,108,109]. This review mainly aims at the pharmacological activity and action mechanism of secondary metabolites derived from mushrooms. 274 secondary metabolites (Figure 1, Figure 2, Figure 3, Figure 4, Figure 5, Figure 6, Figure 7, Figure 8 and Figure 9) from 17 families of mushrooms, including 116 terpenoids, 71 sterols, 22 phenols, 14 polyketides, 9 alkaloids, and some aromatics and pyridines, as well as their biological activities, are reviewed. Among them, terpenoids and sterol-active metabolites accounted for the highest percentage. For example, Terpenoids **27**, **28**, and **65** possess significant anti-tumour activity; **69**–**72**, **126**, **129**–**131**, and **134** show primary anti-inflammatory activity; compounds **35**, **38**, **40**, **82**, **123**, **124**, and **127** possess a significant hypoglycaemic effect. Sterols **147** and **148** exhibit anti-microbial activity, while **176**–**178** exhibit better anti-malarial activity.

In summary, the purpose of the current review is to provide a valuable theoretical reference for researchers to reasonably develop and utilize edible and medicinal mushrooms.

## Figures and Tables

**Table 1:** Different species of mushrooms and their secondary metabolites.

Mushroom	Mushroom Family	Secondary Metabolites	Refs.
*Morchella esculenta*	*Morchellaceae*	**1**–**3**	[13]
*Ganoderma lingzhi*	*Ganodermataceae*	**4**	[14]
*Macrolepiota procera*	*Agaricaceae*	**5**–**16**	[15]
*Fuscoporia torulosa*	*Hymenochaetaceae*	**17**–**26**	[16]
*Phellinus igniarius*	*Polyporaceae*	**27**–**28**	[17]
*Wolfiporia cocos*	*Polyporaceae*	**29**–**46**	[18]
*Ganoderma lingzhi*	*Ganodermataceae*	**47**	[19]
*Calvatia nipponica*	*Lycoperdaceae*	**48**–**52**	[20]
*Butyriboletus roseoflavus*	*Boletaceae*	**53**	[21]
*Ganoderma luteomarginatum*	*Ganodermataceae*	**54**–**56**	[22]
*Pleurotus florida*	*Pleurotaceae*	**57**	[23]
*Laetiporus sulphureus*	*Polyporaceae*	**58**–**59**	[24]
*Sarcosphaera crassa*	*Pezizaceae*	**60**–**63**	[25]
*Tricholoma ustaloides*	*Tricholomataceae*	**64**–**65**	[26]
*Lentinula edodes*	*Omphalotaceae*	**66**–**68**	[27]
*Ganoderma*	*Ganodermataceae*	**69**–**72**	[28]
*Ganoderma lucidum*	*Ganodermataceae*	**73**–**75**	[29]
*Morchella importuna*	*Morchellaceae*	**76**–**78**	[30]
*Oudemansiella raphanipes*	*Tricholomataceae*	**79**–**81**	[31]
*Sanghuangporus sanghuang*	*Hymenochaetaceae*	**82**–**87**	[32]
*Paxillus involutus*	*Paxillaceae*	**88**–**93**	[33]
*Paxillus involutus*	*Paxillaceae*	**94**–**101**	[34]
*Pleurotus ostreatus* and *Pleurotus eryngii*	*Pleurotaceae*	**102**–**103**	[35]
*Morehella importuna*	*Morchellaceae*	**104**–**110**	[36]
*Lepista sordida*	*Tricholomataceae*	**111**–**112**	[37]
*Poria cocos Wolf*	*Polyporaceae*	**113**–**117**	[38]
*Ganoderma lucidum*	*Ganodermataceae*	**118**	[39]
*Fomitopsis pinicola*	*Coriolaceae*	**119**–**127**	[40]
*Ganoderma orbiforme*	*Ganodermataceae*	**128**–**129**	[41]
*Inonotus obliquus*	*Hymenochaetaceae*	**130**–**136**	[42]
*Hericium erinaceus*	*Hericiaceae*	**137**–**138**	[43]
*Laetiporus sulphureus*	*Polyporaceae*	**139**–**141**	[44]
*Ganoderma sinense*	*Ganodermataceae*	**142**–**143**	[45]
*Ganoderma*	*Ganodermataceae*	**144**–**148**	[46]
*Tomophagus* sp.	*Ganodermataceae*	**149**–**163**	[47]
*Hohenbuehelia grisea*	*Pleurotaceae*	**164**–**167**	[48]
*Sanghuangporus*	*Hymenochaetaceae*	**168**–**170**	[49]
*Sanghuangporus*	*Hymenochaetaceae*	**171**–**174**	[50]
*Pleurotus ostreatus* and *Trametes robiniophila*	*Pleurotaceae and Polyporaceae*	**175**	[51]
*Ganoderma*	*Ganodermataceae*	**176**	[52]
*Ganoderma weberianum*	*Ganodermataceae*	**177**–**178**	[53]
*Hericium erinaceus*	*Hericiaceae*	**179**–**180**	[54]
*Ganoderma leucocontextum*	*Ganodermataceae*	**181**–**182**	[55]
*Hericium erinaceus* and *Hericium flagellum*	*Hericiaceae*	**183**–**186**	[56]
*Inonotus hispidus*	*Hymenochaetaceae*	**187**–**192**	[57]
*Ganoderma resinaceum*	*Ganodermataceae*	**193**–**199**	[58]
*Inonotus obliquus*	*Hymenochaetaceae*	**200**	[59]
*Hericium flagellum*	*Hericiaceae*	**201**–**203**	[60]
*Dictyophora indusiata*	*Phallaceae*	**204**–**206**	[61]
*Ganoderma lucidum*	*Ganodermataceae*	**207**–**230**	[62]
*Hericium caput−medusae*	*Hericiaceae*	**231**–**233**	[63]
*Hericium erinaceus*	*Hericiaceae*	**234**–**238**	[64]
*Paxillus involutus*	*Paxillaceae*	**239**–**242**	[65]
*Ganoderma leucocontextum*	*Ganodermataceae*	**243**–**245**	[66]
*Morchella importuna*	*Morchellaceae*	**246**	[67]
*Cordyceps militaris*	*Cordycepitaceae*	**247**–**251**	[68]
*Ganoderma gibbosum*	*Ganodermataceae*	**252**	[69]
*Albatrellus confluens*	*Crypsinus*	**253**–**254**	[70]
*Ganoderma resinaceum*	*Ganodermataceae*	**255**–**264**	[71]
*Pleurotus cornucopiae*	*Pleurotaceae*	**265**–**268**	[72]
*Ganoderma applanatum*	*Ganodermataceae*	**269**	[73]
*Vanderbylia robiniophila*	*Polyporaceae*	**270**–**274**	[74]

## Data Availability

Not applicable.
